# Preface: BITS2014, the annual meeting of the Italian Society of Bioinformatics

**DOI:** 10.1186/1471-2105-16-S9-S1

**Published:** 2015-06-01

**Authors:** Angelo Facchiano, Claudia Angelini, Roberta Bosotti, Alessandro Guffanti, Anna Marabotti, Roberto Marangoni, Stefano Pascarella, Paolo Romano, Andreas Zanzoni, Manuela Helmer-Citterich

**Affiliations:** 1 CNR-ISA, Avellino, Italy; 2 CNR-IAC, Napoli, Italy; 3 Nerviano Medical Sciences, Nerviano (MI), Italy; 4 Genomnia srl, Milano, Italy; 5 University of Salerno, Italy; 6 University of Pisa, Italy; 7 Sapienza University of Rome, Italy; 8 IRCCS San Martino IST, Genoa, Italy; 9 Inserm, UMR1090 TAGC, Marseille, F-13288, France; 10 Aix-Marseille Université, UMR1090 TAGC, Marseille, F-13288, France; 11 University of Rome "Tor Vergata", Italy

## Abstract

This Preface introduces the content of the BioMed Central journal Supplements related to BITS2014 meeting, held in Rome, Italy, from the 26th to the 28th of February, 2014.

## BITS, the Italian Society of Bioinformatics

BITS, the Italian Society of Bioinformatics [1], is the largest non-profit association of researchers involved in Bioinformatics with work activities or interest in Italy. About 300 researchers joined BITS since 2003, when a small number of Italian scientists, working in the field of Bioinformatics, founded this no-profit scientific association, with the aim of diffusing this discipline in Italy. Since its foundation, BITS has continuously increased the number of members and was recognized as a Regional group of the International Society for Computational Biology (ISCB). BITS promoted activities as courses and workshops, at national and international level. Such events were mainly located in Italy and, in some cases, abroad. One of the main goals of the Society is the support to young scientists through travel grants for the participation to international conferences and events of interest for the Bioinformatics community.

## BITS2014 annual meeting

The organization of an Annual Scientific meeting is one of the central activities of BITS. The eleventh BITS meeting has been held in Rome, from the 26th to the 28th of February, 2014. The meeting was organized by Manuela Helmer-Citterich, Elisabetta Pizzi and Anna Tramontano, together with a Scientific Committee including most of the Italian Bioinformatics senior scientists (see Table 1).

**Table 1:** BITS 2014 Program Committee.

Claudia Angelini	CNR, IAC, Napoli, Italy
Marco Antoniotti	University of Milano Bicocca, Italy

Marcella Attimonelli	University of Bari, Italy

Gabriele Ausiello	University of Rome "Tor Vergata", Italy

Riccardo Bellazzi	University of Pavia, Italy

**Roberta Bosotti**	Nerviano Medical Sciences, Nerviano (MI), Italy

Raffaele Calogero	University of Torino, Italy

Nicola Cannata	Next Generation Bioinformatics srl - Camerino (MC), Italy

Rita Casadio	University of Bologna, Italy

Maria Luisa Chiusano	University of Naples Federico II, Napoli, Italy

Francesca Ciccarelli	King's College London, UK

Francesca Cordero	University of Torino, Italy

Susan Costantini	INT Pascale - CROM, Mercogliano (AV), Italy

Domenica D'Elia	CNR, Institute for Biomedical Technologies, Bari, Italy

**Angelo Facchiano**	CNR, Institute of Food Science, Avellino, Italy

Alfredo Ferro	University of Catania, Italy

Federico Fogolari	University of Udine, Italy

Carmela Gissi	University of Milan, Italy

Rosalba Giugno	University of Catania, Italy

Giorgio Grillo	CNR, Institute for Biomedical Technologies, Bari, Italy

**Alessandro Guffanti**	Genomnia srl, Milano, Italy

**Manuela Helmer-Citterich**	University of Rome "Tor Vergata", Italy

Giovanni Lavorgna	Ospedale San Raffaele, Milan, Italy

Vito Flavio Licciulli	CNR, Institute for Biomedical Technologies, Bari, Italy

Sabino Liuni	CNR, Institute for Biomedical Technologies, Bari, Italy

Alberto Magi	University of Firenze, Italy

Paolo Magni	University of Pavia, Italy

**Anna Marabotti**	University of Salerno, Italy

**Roberto Marangoni**	University of Pisa, Italy

Marco Masseroli	Politecnico di Milano, Italy

Giancarlo Mauri	University of Milano Bicocca, Italy

Cristian Micheletti	SISSA, Scuola Internazionale Superiore di Studi Avanzati, Trieste, Italy

Veronica Morea	CNR -Institute of Molecular Biology and Pathology, Rome, Italy

Marco Muselli	CNR - Institute of Electronics, Computer and Telecommunication Engineering, Genoa, Italy

Alessandro Pandini	King's College London, UK

**Stefano Pascarella**	Sapienza University of Rome, Italy

Marco Pellegrini	CNR - Istituto di Informatica e Telematica, Pisa, Italy

Graziano Pesole	CNR - Institute of Biomembranes and Bioenergetics, Bari, Italy

Ernesto Picardi	University of Bari, Italy

Elisabetta Pizzi	Istituto Superiore di Sanità, Rome, Italy

Alberto Policriti	University of Udine, Italy

Uberto Pozzoli	IRCCS 'E.Medea', Bosisio Parini (LC), Italy

Alfredo Pulvirenti	University of Catania, Italy

**Paolo Romano**	IRCCS San Martino IST, Genoa, Italy

Remo Sanges	Stazione Zoologica "Anton Dorhn", Napoli, Italy

Roberto Tagliaferri	University of Salerno, Italy

Silvio C.E. Tosatto	University of Padova, Italy

Anna Tramontano	Sapienza University of Rome, Italy

Luigi Varesio	Giannina Gaslini Institute, Genoa, Italy

Saverio Vicario	CNR, Institute for Biomedical Technologies, Bari, Italy

Nicola Vitulo	University of Padova, Italy

Stefano Volinia	University of Ferrara, Italy

**Andreas Zanzoni**	TAGC U1090, Inserm, Marseille, France

The names of the Associate Editors of this Supplements are in bold.

About 200 participants attended the meeting, the highest number over the past BITS meetings. The scientific program included three keynote speakers: Ewan Birney, Joint Associate Director and Senior Scientist at EBI, Gunnar von Heijne, Dept. of Biochemistry and Biophysics - Stockholm University, and Franca Fraternali, Group Leader at King's College London.

Participants were invited to submit scientific contributions either as oral presentations or posters. After evaluation of the abstracts, the Scientific Committee selected 29 proposals for oral presentations, and accepted 120 posters.

The topics of the meeting initially were not restricted to specific areas of interest in Bioinformatics. However, after the selection of the abstracts, the Conference program was organized into the following sessions: Omics; Synthetic and Systems Biology; Algorithms for Bioinformatics; Macromolecular Structure; Stochastic Modeling; Biological Databases; Genomics, Transcriptomics and NGS; Management Integration and Analysis of Clinical and Biomolecular Data; Metagenomics; Molecular Evolution; Pharmacogenomics; Protein Structure and Function; Proteomics.

## BITS2014: supplements to BioMed Central journals

All the Authors of the scientific contributions that were presented at the meeting in form of either oral presentations or posters were invited, after the meeting, to prepare and submit a manuscript to be evaluated for publication in a BioMed Central journal Supplement.

The Editor of the Supplement, Angelo Facchiano, designated by the Bioinformatics Italian Society Steering Committee, invited some of the Scientific Committee members to serve as Associate Editors (see names highlighted in bold characters in Table 1) for the handling of the submitted manuscripts. Manuscripts were peer-reviewed in agreement with BMC rules for manuscripts' Supplements evaluation, and 20 out of 25 originally submitted manuscripts were selected.

## Evaluation process outline

As for previous BioMed Central journal supplements for BITS meetings [2], a peer-reviewed careful evaluation of manuscripts has been applied. Each manuscript was assigned to an Associate Editor, on the basis of specific expertise and, when possible, by considering the previous involvement in the contributions selection process. Any conflict of interest was of course avoided.

The Associate Editor preliminarily evaluated the manuscript and assigned it to three independent reviewers, selected from international literature or personal contact, on the basis of their specific expertise. In few cases, the third evaluation was directly performed by the Associate Editor or by another Associate Editor with suitable expertise.

In case of Reviewers' negative comments or if the manuscript was not sent to peer review per Associate Editor decision, the manuscript was rejected.

In case of favorable Reviewers evaluations, their comments were sent to the Authors with the request of submitting a major/minor revised version of their manuscript within two months. The revised version of the manuscript was then sent for a further evaluation to the reviewers.

In case only minor revision was recommended, the Supplement Editor allowed the Associate Editor to decide the suitability for publication without sending it again to the reviewers, also in consideration of the given reviewer availability to re-evaluate the manuscript.

In few cases, a further revised manuscript was requested to the Authors with subtle modifications, in agreement to reviewers' comments.

At the end of this process, 20 articles were accepted and included in this supplement. The articles are published as supplements in BMC Bioinformatics, BMC Genomics, and BMC Systems Biology.

A short presentation of each of these contributions is shown below.

## Bioinformatics

Seven articles have been accepted for publication in BMC Bioinformatics. They cover different aspects of the field, from novel algorithms, application and comparison of analysis methods to specific data, tool development.

Carrara et al. [3] investigate the relationship between different Library Sample Preparation methods and bioinformatics analysis methods on the efficiency of the detection and quantification of alternative splicing from paired end datasets (50 bp). Their interesting and somewhat surprising results allow to discriminate among protocols for alternative splicing detection. The analyses performed with transcript-focused and exon-focused methods show comparable results, with a better performance of exon-level methods for higher sequence depth, as measured by spike-ins. Moreover, the lower sensitivity in detection of isoforms is associated with the rRNA depletion as opposed to PolyA^+^ selection. This would be due to the sequence reads used for non-coding RNA (supposedly long ncRNAs) coverage, which would be under represented in PolyA^+^ libraries. Finally, all the test datasets are made available to the community, including the spike-ins. This will be important for method developers.

Supervised classification is a well-known problem in statistical learning. From one hand, there is a great amount of methods that perform the classification as "black-box" (i.e., without providing a set of intelligible rules explaining how the classification is carried out). On the other hand, there are relatively few approaches that provide intelligible "AND-type" and "OR-type" rules. Parodi et al. [4] illustrate the use of Logic Learning Machine (LLM) applied to the differential diagnosis of pleural mesothelioma. The interest of the paper is in the use of LMM, an innovative method of supervised data analysis, capable of building classifiers described by a set of intelligible rules including simple conditions in their antecedent part. The use of intelligible rule is of great interest in biomedical applications. Unfortunately, in many cases such rules have poor accuracy. Here, the Authors show that LMM has good performance when compared with classical data mining methods, suggesting that the proposed approach can be used in more complex contexts.

Prezza and Policriti [5] present a read mapper and aligner tool called BW-ERNE. Their work shows that indexes based on de Bruijn hash functions can be compressed using the BWT technique, and so BW-ERNE is an elegant solution to compress the index for approximate string matching using a hash function which is both Hamming and de Bruijn-aware.

Calabria et al. [6] describe the development of a LIMS system, named adLIMS, which tracks cell samples collected by clinicians as soon as they are processed, using PCR and Next Generation Sequencing methods, to produce data for research projects and clinical trials. The system was developed by modifying an existing open source software, ADempiere ERP, in order to satisfy the necessary user requirements.

Atomistic molecular dynamics simulations with explicit solvent, coupled to enhanced sampling techniques and advanced analysis methods, allow to study at molecular level several fundamental biological *phenomena*, such as the regulation of gene expression. In the paper by Di Palma et al. [7] these approaches are used to study the structural mechanism regulating the conformational changes of bacterial riboswitches upon binding of sensed ligands, suggesting a molecular explanation of these phenomena. Results are qualitatively in agreement with experimental data, but this study dissected the influence of different protocols used for simulations, highlighting the need for more advanced techniques in order to allow a quantitative interpretation of the results.

Pio et al. [8] describe a database and a web tool for the *consensus *analysis of miRNA-mRNA interactions, a resource that is very useful since such interaction data is noisy and difficult to explore. The tool integrates the predictions that are provided by some known tools. Then it runs an ensemble classifier on the data with the aim of optimizing results. The work also compares ComiRNet with similar tools, showing its superiority in terms of better false positives and false negatives rates.

Caravagna et al. [9] describe in their "Software" article a Python-based package, intended for public release, useful to analyze time series signals, and specifically designed for Systems Biology data.

## Genomics

Fiscon and colleagues [10] present MONSTER, a novel software for the detection of structural motifs in RNA, in particular in long non-coding RNA (lncRNA) and non-branching structures. In this work, structural motifs are searched taking into account also sub-optimal solutions. The Authors describe the procedure and its application to HOTAIR, ANRIL and COLDAIR, three lncRNAs that are all known to interact with the same protein complexes of the Polycomb group.

The analysis of RNA-seq data has received great attention in the last few years. Nowadays, RNA-seq is becoming the most widely used approach for studying gene expression. However, despite an increasing number of experiments for studying the dynamics of gene expression changes in cells in response to *stimuli *and environmental conditions, there is still a lack of computational methods useful for analyzing such data in the context of time course experiments. The work of Sanavia et al. [11] represents one of the few methods available. The FunPat approach combines functional annotations, clustering and gene selection and allows one to identify differentially expressed (DE) genes from time-course RNA-seq experiments. In particular, the method provides clusters of DE genes associated to temporal patterns and specific biological terms. The application to two real datasets confirms the ability of FunPat to produce results with high reproducibility on different time series expression data. In the near future, thanks to the decreasing cost of sequencing, it is very likely that time course experiments with RNA-seq will become routinely used to study gene expression dynamics.

D'Antonio et al. [12] propose a novel pipeline for the cloud-based analysis of RNA-seq data, called RAP (RNA-seq Analysis Pipeline). RAP has been implemented as a combination of analysis modules, which cover the most common applications of RNA-seq data. Since it is offered as a cloud-based web application, it is especially attractive for novice users or for researchers that might not have access to the necessary computational resources. However, the provided flexibility in terms of configuration options also makes it useful for advanced users.

Martignetti et al. [13] describe a new intuitive computational approach to infer cancer-related regulatory activity of miRNAs on transcriptome, by combining miRNA and mRNA expression data with sequence-based target predictions. The Authors apply the method to breast cancer samples and identify potentially interesting miRNA-target correlations, in particular in the triple negative tumor subtype. In the latest years, literature reports indicating deregulation of miRNA expression in many tumors have strongly stimulated miRNA research field. In this scenario, the described approach constitutes an additional tool that could support the investigation of the role of miRNA in cancer development.

Masseroli et al. [14] describe the Genomic and Proteomic Knowledge Base (GPKB), a global bioinformatics repository to detect and complement biomolecular annotations and candidate protein-protein interactions (PPI)-associated disorders. GPKB includes many distributed biomolecular annotation and interaction data of several organisms, such as 1,566,842 gene annotations, 91,581,198 protein annotations and 209,674 PPIs.

## Systems Biology

Fornari et al. [15] implemented an extended version of a mathematical model to study Cancer Stem Cells (CSCs)-based cancer progression. This enhanced model takes into account the tumor microenvironment, by including the cell subpopulations of CSCs-based tumors, and is implemented in a novel workflow for the characterization of complex system behaviors. Interestingly, the proposed approach can be easily generalized and may be used to study other dynamic systems.

The paper by Sin et al. [16] faces a very important problem among cellular control mechanisms: the RNA degradation. In this paper, the Authors, on the basis of a deep data analysis on single RNA molecule degradation, propose an innovative degradation pathway that, by introducing new intermediate stages, can account for the major discrepancies observed between the existing degradation models and the experimental data recorded.

The work by Galeota et al. [17] contributes to the debate about protein interaction network simulation, an emerging field of the biological research as demonstrated by the number of papers dealing with this issue in the recent scientific literature. The Authors tried to take advantage of the accumulating experimental information about molecular cell components to ask fundamental questions regarding the topological properties of the cell interactome and their influence on protein network self-organization. The work provides the computerized model ProtNet to simulate protein network and proposes two quantitative parameters, namely Self Similarity Index and Inter Cells Similarity Index, to measure the self-organization within one pseudo-cell and to compare network organization in two different cells, respectively. These parameters may be of general use in the field.

The article by Alaimo and coworkers [18] describes a freely available, Web-based application that performs predictions on drug-target interactions and drug combination, allowing users to retrieve information on drugs and targets, or to perform predictions based on users' own data. The field of drug development has become more and more challenging in the last years, because of the increasing costs related to the discovery of novel molecules with therapeutic activity. Therefore, bioinformatics approaches able to optimize the efforts to find effective therapeutic agents, such as the one described in this paper, are of invaluable importance for all people involved in drug discovery and design.

The paper "A knowledge base for the Vitis vinifera functional analysis" by Pulvirenti et al. [19] presents a bioinformatics resource for the analysis of grapevine genomics data, BioWine. Its main added value resides in its ability to integrate RNA-seq experiments and in its connection to many external databases, which also allows users to exploit gene set enrichment. The web interface is very friendly and effective, so that the platform can be a useful tool for data analysis by end-users and it can become a standard interface for the analysis of genomes of agricultural interest.

Politi et al. [20] present a work on the analysis of the effect of cell-to-cell variability in the parameter estimation from experimental data, in the context of synthetic biology. This work provides an analysis of how various kinds of noise affect input-output function of gene regulatory circuits, with special care to potential modularity of the circuits, i.e., to analyze context-dependent variability of module function under noise environment.

Paradisi et al. [21] investigate super-concentration of solutes during spontaneous liposome formation. The paper directly addresses experimental observations made by Luisi and co-workers in 2010 [22] and indicated as the "super concentration effect". The mathematical model is based on Cox's Renewal Theory. Its application to the protein accumulation inside vesicles requires a cooperativity between protein and vesicle plus some additional conditions that allow to view the system as independent vesicles interacting with a reservoir of proteins. Then, a power-law distribution is obtained by a protein-induced slowing down of the vesicle-closing rate at constant influx of proteins into the open vesicles.

Modeling biochemical reactions as stochastic processes allows one to simulate experiments able to reproduce the behavior of real biochemical systems. Most of reactions are modeled as discrete-state continuous-time stochastic processes evolving as memory-less Markov processes. However, in some cases, it has been observed that biochemical systems exhibit non-Markovian dynamics. Classical simulation approaches are not able to capture such dynamics. Chiarugi et al. [23] propose an algorithm for building stochastic simulations that models more precisely non-Markovian processes in some specific situations. They show that their algorithm can obtain accurate models of enzymatic reactions that, in specific cases, fit experimental data better than the corresponding Markovian models.

## Competing interests

The authors declare that they have no competing interests.

## Declarations

This supplement has not been supported by sponsorship.

This article has been published as part of *BMC Bioinformatics *Volume 16 Supplement 9, 2015: Proceedings of the Italian Society of Bioinformatics (BITS): Annual Meeting 2014: Bioinformatics. The full contents of the supplement are available online at http://www.biomedcentral.com/bmcbioinformatics/supplements/16/S9.

## References

[B1] Italian Society of Bioinformatics http://www.bioinformatics.it/

[B2] Gissi C Romano P Ferro A Giugno R Pulvirenti A Facchiano A Helmer-Cittierch M Bioinformatics in Italy: BITS2012, the ninth annual meeting of the Italian Society of Bioinformatics BMC Bioinformatics 2014 14 S1 23815154 10.1186/1471-2105-14-S7-S1 PMC3633006

[B3] Carrara M Lum J Cordero F Beccuti M Poidinger M Donatelli S Calogero RA Zolezzi F Alternative splicing detection workflow needs a careful combination of sample prep and bioinformatics analysis BMC Bioinformatics 2015 16 Suppl 9 S2 26050971 10.1186/1471-2105-16-S9-S2 PMC4464605

[B4] Parodi S Filiberti R Marroni P Libener R Ivaldi GP Mussap M Ferrari E Manneschi C Montani E Muselli M Differential diagnosis of pleural mesothelioma using Logic Learning Machine BMC Bioinformatics 2015 16 Suppl 9 S3 26051106 10.1186/1471-2105-16-S9-S3 PMC4464205

[B5] Prezza N Policriti A Fast randomized approximate string matching with succinct hash data structures BMC Bioinformatics 2015 16 Suppl 9 S4 26051265 10.1186/1471-2105-16-S9-S4 PMC4464037

[B6] Calabria A Spinozzi G Benedicenti F Tenderini E Montini E adLIMS: a customized open source software that allows bridging clinical and basic molecular research studies BMC Bioinformatics 2015 16 Suppl 9 S5 26051409 10.1186/1471-2105-16-S9-S5 PMC4464029

[B7] Di Palma F Bottaro S Bussi G Kissing loop interaction in adenine riboswitch: insights from umbrella sampling simulations BMC Bioinformatics 2015 16 Suppl 9 S6 26051557 10.1186/1471-2105-16-S9-S6 PMC4464220

[B8] Pio G Ceci M Malerba D D'Elia D ComiRNet: a web-based system for the analysis of miRNA-gene regulatory networks BMC Bioinformatics 2015 16 Suppl 9 S7 26051695 10.1186/1471-2105-16-S9-S7 PMC4464030

[B9] Caravagna G De Sano L Antoniotti M Automatising the analysis of stochastic biochemical time-series BMC Bioinformatics 2015 16 Suppl 9 S8 26051821 10.1186/1471-2105-16-S9-S8 PMC4464019

[B10] Fiscon G Paci P Iannello G MONSTER v1.1: a tool to extract and search for RNA non-branching structures BMC Genomics 2015 16 Suppl 6 S1 26047478 10.1186/1471-2164-16-S6-S1 PMC4460781

[B11] Sanavia T Finotello F Di Camillo B FunPat: function-based pattern analysis on RNA-seq time series data BMC Genomics 2015 16 Suppl 6 S2 26046293 10.1186/1471-2164-16-S6-S2 PMC4460925

[B12] D'Antonio M D'Onorio De Meo P Pallocca M Picardi E D'Erchia AM Calogero R Castrignanò T Pesole G RAP: RNA-Seq Analysis Pipeline, a new cloud-based NGS web application BMC Genomics 2015 16 Suppl 6 S3 26046471 10.1186/1471-2164-16-S6-S3 PMC4461013

[B13] Martignetti L Tesson B Almeida A Zinovyev A Tucker GC Dubois T Barillot E Detection of miRNA regulatory effect on triple negative breast cancer transcriptome BMC Genomics 2015 16 Suppl 6 S4 26046581 10.1186/1471-2164-16-S6-S4 PMC4460783

[B14] Masseroli M Canakoglu A Quigliatti M Detection of gene annotations and protein-protein interaction associated disorders through transitive relationships between integrated annotations BMC Genomics 2015 16 Suppl 6 S5 26046679 10.1186/1471-2164-16-S6-S5 PMC4460591

[B15] Fornari C Balbo G Halawani SM Ba-Rukab O Rahman Ahmad A Calogero RA Cordero F Beccuti M A versatile mathematical workflow to explore how Cancer Stem Cell fate infuences tumor progression BMC Systems Biology 2015 9 Suppl 3 S1 26050594 10.1186/1752-0509-9-S3-S1 PMC4464028

[B16] Sin C Chiarugi D Valleriani A Single-molecule modeling of mRNA degradation by miRNA: Lessons from data BMC Systems Biology 2015 9 Suppl 3 S2 26050661 10.1186/1752-0509-9-S3-S2 PMC4464216

[B17] Galeota E Gravila C Castiglione F Bernaschi M Cesareni G The hierarchical organization of natural protein interaction networks confers self-organization properties on pseudocells BMC Systems Biology 2015 9 Suppl 3 S3 26050708 10.1186/1752-0509-9-S3-S3 PMC4464023

[B18] Alaimo S Bonnici V Cancemi D Ferro A Giugno R Pulvirenti A DT-Web: a web-based application for Drug-Target interaction and drug combination prediction through domain-tuned network-based inference BMC Systems Biology 2015 9 Suppl 3 S4 26050742 10.1186/1752-0509-9-S3-S4 PMC4464606

[B19] Pulvirenti A Giugno R Distefano R Pigola G Mongiovi M Giudice G Vendramin V Lombardo A Cattonaro F Ferro A A knowledge base for the Vitis vinifera functional Analysis BMC Systems Biology 2015 9 Suppl 3 S5 26050794 10.1186/1752-0509-9-S3-S5 PMC4464603

[B20] Politi N Pasotti L Zucca S Magni P Modelling the effects of cell-to-cell variability on the output of interconnected gene networks in bacterial populations BMC Systems Biology 2015 9 Suppl 3 S6 26050995 10.1186/1752-0509-9-S3-S6 PMC4464218

[B21] Paradisi P Allegrini P Chiarugi D A renewal model for the emergence of anomalous solute crowding in liposomes BMC Systems Biology 2015 9 Suppl 3 S7 26051120 10.1186/1752-0509-9-S3-S7 PMC4464207

[B22] Luisi PL Allegretti M Pereira de Souza T Steiniger F Fahr A Stano P Spontaneous protein crowding in liposomes: A new vista for the origin of cellular metabolism ChemBioChem 2010 11 1989 1992 10.1002/cbic.201000381 20806308

[B23] Chiarugi D Falaschi M Hermith D Olarte C Torella L Modelling non-Markovian Dynamics in Biochemical Reactions BMC Systems Biology 2015 9 Suppl 3 S8 26051249 10.1186/1752-0509-9-S3-S8 PMC4464211

